# Identification of novel, therapy-responsive protein biomarkers in a mouse model of Duchenne muscular dystrophy by aptamer-based serum proteomics

**DOI:** 10.1038/srep17014

**Published:** 2015-11-23

**Authors:** Anna M. L. Coenen-Stass, Graham McClorey, Raquel Manzano, Corinne A. Betts, Alison Blain, Amer F. Saleh, Michael J. Gait, Hanns Lochmüller, Matthew J. A. Wood, Thomas C. Roberts

**Affiliations:** 1 Department of Physiology, Anatomy and Genetics, University of Oxford, South Parks Road, Oxford, OX1 3QX, UK; 2 The John Walton Muscular Dystrophy Research Centre, MRC Centre for Neuromuscular Diseases, Institute of Genetic Medicine, Newcastle University, Central Parkway, Newcastle upon Tyne, NE1 3BZ, UK; 3 Medical Research Council, Laboratory of Molecular Biology, Francis Crick Avenue, Cambridge, CB2 0QH, UK; 4 Sanford Burnham Prebys Medical Discovery Institute, Development, Aging and Regeneration Program, La Jolla, CA, 92037, USA

## Abstract

There is currently an urgent need for biomarkers that can be used to monitor the efficacy of experimental therapies for Duchenne Muscular Dystrophy (DMD) in clinical trials. Identification of novel protein biomarkers has been limited due to the massive complexity of the serum proteome and the presence of a small number of very highly abundant proteins. Here we have utilised an aptamer-based proteomics approach to profile 1,129 proteins in the serum of wild-type and *mdx* (dystrophin deficient) mice. The serum levels of 96 proteins were found to be significantly altered (*P* < 0.001, *q* < 0.01) in *mdx* mice. Additionally, systemic treatment with a peptide-antisense oligonucleotide conjugate designed to induce *Dmd* exon skipping and recover dystrophin protein expression caused many of the differentially abundant serum proteins to be restored towards wild-type levels. Results for five leading candidate protein biomarkers (Pgam1, Tnni3, Camk2b, Cycs and Adamts5) were validated by ELISA in the mouse samples. Furthermore, ADAMTS5 was found to be significantly elevated in human DMD patient serum. This study has identified multiple novel, therapy-responsive protein biomarkers in the serum of the *mdx* mouse with potential utility in DMD patients.

Duchenne Muscular Dystrophy (DMD) is a lethal monogenic disorder and the most prevalent inherited myopathy affecting children. Although currently incurable, many therapeutic strategies for DMD have been proposed. A highly promising approach is antisense oligonucleotide-mediated exon skipping, which aims to modulate splicing of the dystrophin gene (human: *DMD*, mouse: *Dmd*) so as to restore the translation reading frame which is disrupted by DMD-associated mutations. We have developed a second generation exon skipping technology called PPMO (peptide-phosphorodiamidate morpholino oligonucleotide) which induces exon skipping and dystrophin restoration at high levels in skeletal muscle and diaphragm, and at moderate levels in heart^1^,^2^. Clinical trials with first generation exon skipping compounds (2′-*O*-methyl RNA and naked PMO chemistry) have demonstrated both promise^3^,^4^ and some disappointment^5^. One of the difficulties in assessing the effectiveness of these experimental therapies has been finding appropriate outcome measures of clinical benefit.

Methods for assessing the restoration of dystrophin protein in treated patients are currently restricted to analyses of muscle biopsies (e.g. western blot and immunofluorescence microscopy). Conceptually, the usefulness of muscle biopsies is limited, given that only a tiny fraction of a single muscle is assayed, using only semi-quantitative measures. Muscle biopsy is also highly invasive and painful for patients, meaning that serial measurements are not ethically permissible or practical. Similarly, the six minute walk test, in which the distance walked by a patient within 6 minutes is recorded before and after treatment^6^, also has limitations including high inter-patient variability and an unclear relationship to disease natural history. The usefulness of this test may also be limited by the patient’s compliance with the protocol. Furthermore, some patients lose ambulation as a result of the natural progression of the disease, meaning that they are unable to perform the test. An alternative approach has been to use a panel of functional outcome measures such as The North Star Ambulatory Assessment (NSAA) protocol^7^. However, NSAA is also only applicable to ambulant patients, thereby excluding those patients with the most severe disease manifestation.

Magnetic Resonance Imaging (MRI) is another method of monitoring pathology in DMD by revealing the extent of muscle degeneration (i.e. muscle wasting, fibrosis and deposition of adipose tissue) but does not provide a direct read-out of muscle function *per se*. Additionally, this approach is subject to a number of limitations such as high cost, low through-put and a requirement for specialist personnel trained in interpreting the MRI data. MRI is therefore less suitable for use in trials with large patient cohorts than clinical chemistry analysis.

Elevated serum creatine kinase (CK) is commonly used as a clinical chemistry biomarker for neuromuscular disorders, and DMD in particular. However, serum CK levels vary widely in healthy individuals and may also be elevated in asymptomatic individuals. In addition, serum CK is sensitive to a number of factors such as exercise, age, race, and pharmacological interventions (e.g. statin use). CK is also considered a non-specific marker of muscle damage as it is elevated after myocardial infarction, myocarditis, myositis, rhabdomyolysis and rhabdomyosarcoma^8^. While hyperCKemia is indicative of DMD-associated muscle damage, serum CK levels may decline in older patients with more advanced disease on account of reduced muscle mass^9^. Importantly, serum CK measurements do not always correlate with other read-outs of muscle pathology such as MRI, thus they are of limited value for monitoring the effectiveness of experimental therapies^10^.

Consequently, there is an urgent need for minimally-invasive biomarkers that can be used as outcome measures in pre-clinical studies and clinical trials for DMD therapeutics. Analysis of blood is frequently the first line of clinical investigation as it is simple, safe and provides insight into the physiology of the entire body. One source of possible biomarkers is circulating microRNAs^11^. We and others have shown that muscle-specific microRNAs are enriched in the serum of dystrophic animal models^12^,^13^,^14^ and in DMD patients^15^. However, protein-based biomarkers are perhaps preferable on account of the ease with which they can be integrated into existing clinical biochemistry workflows (i.e. measurement by Enzyme-Linked Immunosorbent Assay (ELISA) or standard colourimetric/turbidimetric assays).

The search for novel protein biomarkers entails the application of proteomics techniques to biofluids such as serum or plasma. Mass spectrometry techniques are by far the most commonly used methods of whole proteome analysis and recent developments have enabled the simultaneous measurement of over 10,000 proteins from cell culture lysates^16^ and over 3,000 proteins in dystrophic mouse muscle^17^. However, proteomic analysis of the serum/plasma proteome is complicated by the immense complexity of these biofluids. In principle, the circulation contains every possible protein in the body as it consists of both secreted proteins which exercise their functions in the extracellular space, and proteins derived from tissues that leak into the bloodstream following injury or stress. Furthermore, the composition of the circulating proteome is highly uneven, with a group of ~20 highly abundant proteins comprising ~90% of the total protein (e.g. serum albumin, α2-macroglobulin, immunoglobulins, fibrinogen etc.) Consequently, the majority of circulating proteins (including potential biomarkers) are typically much less abundant. Extracellular proteins span a concentration range of ~10 logs, meaning that detection of lowly abundant potential biomarker proteins is frequently masked by massively more abundant proteins^18^. As a result of these difficulties, mass spectrometric methods generally perform poorly on complex protein mixtures derived from biofluids.

To overcome these challenges we have utilised an aptamer-based affinity purification methodology (SOMAscan) to analyse the serum proteome of wild-type, dystrophic and PPMO-treated mice. This technology enables the determination of protein concentrations for a specific set of targets independent of highly abundant serum proteins which confound mass spectrometry based analyses.

The use of genetically homogenous animal models is advantageous on account of low inter-animal variation. As a result, statistical power to detect differences is high despite relatively small sample sizes. Using this experimental approach we have identified multiple novel therapy-responsive protein biomarkers in murine serum. One of the most promising candidate biomarkers, ADAMTS5, was further validated in DMD patient serum.

## Results

### Proteomic profiling of dystrophic serum by SOMAscan

To identify novel protein biomarkers for DMD we harvested serum from 14 week old C57, *mdx* and PPMO-treated *mdx* mice (*n* = 8). Treated mice were injected at 12 weeks of age with a single 12.5 mg/kg dose of Pip6a-PMO and harvested 2 weeks later as described previously^1^,^13^. Dystrophin protein restoration in quadriceps femoris muscles of treated animals was confirmed by western blot, and the degree of *Dmd* exon 23 skipping determined by RT-qPCR (Supplementary Fig. S1a,b). Median Dmd protein expression in Pip6a-PMO-treated animals was 39% that of wild-type controls, which is comparable to levels observed in our previous studies^1^,^13^. Western blot and RT-qPCR data were strongly correlated (Supplementary Fig. S1c).

Serum protein abundance was profiled using the SOMAscan platform^19^, an affinity-capture based approach which consists of 1,129 SOMAmers (Slow Off-rate Modified Aptamers) designed to bind to human serum proteins (the majority of which exhibit reactivity, or predicted reactivity based on homology, to their murine homologues). SOMAmer-protein complexes are precipitated and protein concentration inferred by hybridising the SOMAmers to DNA micorarrays. To increase the dynamic range of analyte detection, the SOMAscan methodology utilises three dilutions of serum samples (0.5%, 2% and 5% respectively) with a unique set of SOMAmer reagents used to detect target proteins at each dilution level^19^.

Applying this approach, we were able to detect all SOMAmer targets in all samples. One treated sample (T7) was excluded from the analysis as it did not pass quality control checks for biases in SOMAmer hybridization (Supplementary Fig. S2a). Probe hybridization was equivalent between all samples (Supplementary Fig. S2b) although median normalisation scale factors were slightly skewed in the *mdx* samples, suggesting an increase in total protein concentration in these samples (Supplementary Fig. S2c). This observation is similar to the increase in total RNA and total microRNA in dystrophic serum we have reported previously^20^. Experimental groups were clearly separated by unsupervised clustering analysis (Supplementary Fig. S3a) and principal component analysis (Supplementary Fig. S3b) suggesting that serum protein measurements can be utilised to distinguish dystrophic from healthy individuals.

**Figure 1:**
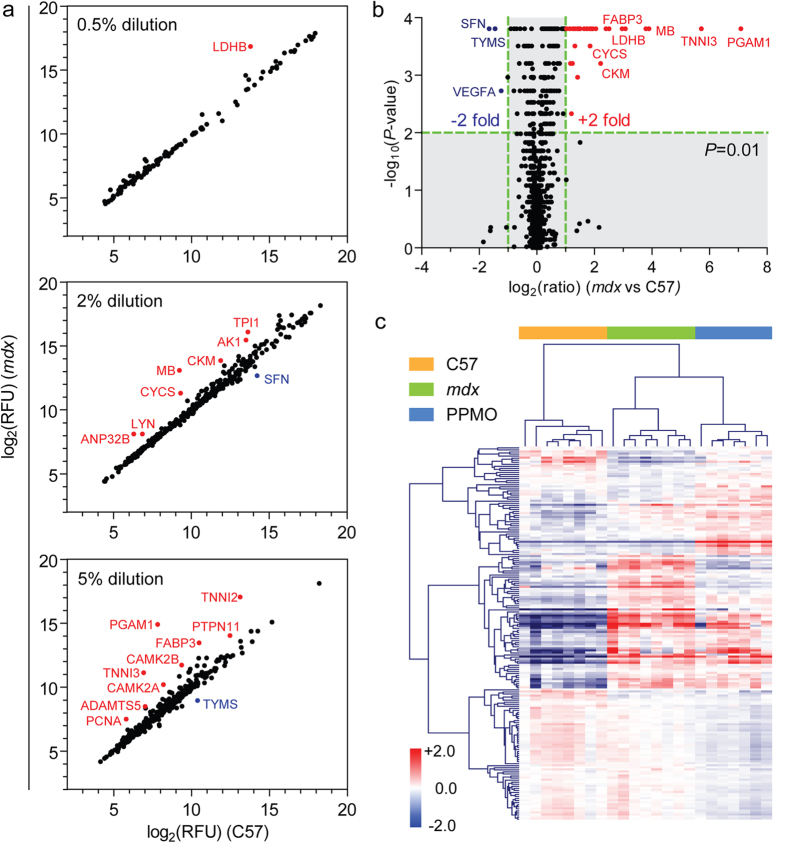
Identification of novel DMD biomarkers. Serum samples from C57, *mdx* and Pip6a-PMO-treated *mdx* were analysed using the SOMAscan methodology. (**a**) Scatter plot of mean relative fluorescent units (RFU) for *mdx* vs C57 identifies differentially abundant proteins separated by dilution group. (**b**) Statistically significant (*P* < 0.01) protein changes in *mdx* serum were determined by Mann-Whitney U test and visualized by volcano plot. (**c**) Proteins with statistically significant changes when comparing between all three experimental groups (Kruskal-Wallis one-way ANOVA *P* < 0.001, *q* < 0.01) were analysed by hierarchical clustering. Red indicates more abundant proteins and blue indicates less abundant proteins. The scale bar represents the row z-score.

Comparison of the C57 and *mdx* groups revealed 96 proteins with statistically significant changes in abundance (Mann-Whitney U test, FDR correction, *P* < 0.001, *q* < 0.01) of which 75 showed an increase and 21 a decrease in *mdx* sera. Highly differentially abundant proteins at each dilution level were visualised by scatter plot (Fig. 1a). The majority of differentially abundant proteins were observed in the higher dilution samples (i.e. lower abundant proteins) with only LDHB (lactate dehydrogenase B) identified in the 0.5% dilution group (i.e. highly abundant proteins) (Fig. 1a). Proteins that were both highly differentially expressed and highly statistically significant were identified by volcano plot (Fig. 1b).

When all three experimental groups were compared, 130 proteins were identified as statistically different (Kruskal-Wallis one-way ANOVA, *P* < 0.001, *q* < 0.01) of which 104 were restored towards wild-type levels after PPMO treatment (73 proteins were more, and 31 less abundant in *mdx* sera respectively). Experimental groups were well separated by hierarchical clustering (Fig. 1c) and principal component analysis (Supplementary Fig. S4a). Substantial overlap was observed between the proteins found to be significant by Mann-Whitney U test and Kruskal-Wallis one-way ANOVA (Supplementary Fig. S4b).

**Figure 2:**
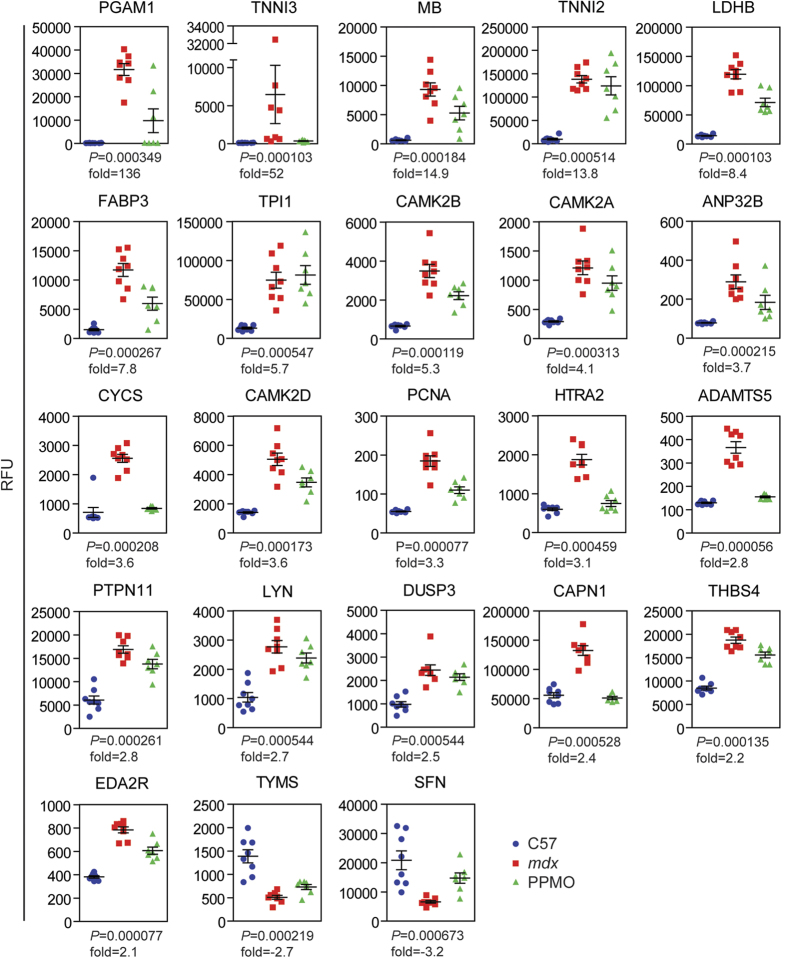
Top candidate disease biomarkers in dystrophic and exon skipping-treated serum. Plots of protein abundance data showing each individual biological replicate for the top 23 ranked candidate biomarkers identified by SOMAscan. Error bars indicate mean +/− SEM. Kruskal-Wallis one-way ANOVA *P* values and *mdx* vs C57 fold changes are indicated for each protein. *q* = 0.00822 for all proteins shown.

A set of 21 proteins with increased and 2 with reduced serum abundance were identified as the most promising candidate DMD biomarkers based on the following criteria: (i) differentially abundant in *mdx* serum by >2 fold, (ii) statistically significant (*P* < 0.01) as determined by both Kruskal-Wallis one-way ANOVA and Mann-Whitney U test, (iii) proteins which responded to treatment with Pip6a-PMO were prioritised (Fig. 2, Table 1). Response to therapy was defined as a shift in the mean protein abundance towards wild-type levels. Some proteins showed complete normalisation (i.e. CYCS, ADAMTS5, HTRA2 and CAPN1), whereas the majority of proteins showed more modest restoration (i.e. MB, LDHB, FABP3, CAMK2B). Two proteins (TNNI2 and TPI1) that were among the most differentially abundant proteins in *mdx* sera displayed little or no response to therapy but were included based on their potential as diagnostic biomarkers.

This list contains a number of novel potential DMD biomarkers and some proteins which have been identified previously (i.e. MB, LDHB, FABP3, CYCS, TPI1 and THBS4)^21^,^22^,^23^. Interestingly, many of these proteins are associated with known pathophysiological features of DMD including muscle function (MB, TNNI2, TNNI3), metabolic dysregulation (PGAM1, LDHB, TPI1, FABP3), calcium metabolism (CAMK2A, CAMK2B, CAMK2C, CAPN1), and extracellular matrix remodelling/fibrogenesis (ADAMTS5, THBS4)^24^.

### ELISA validation of candidate biomarkers in murine serum

**Figure 3:**
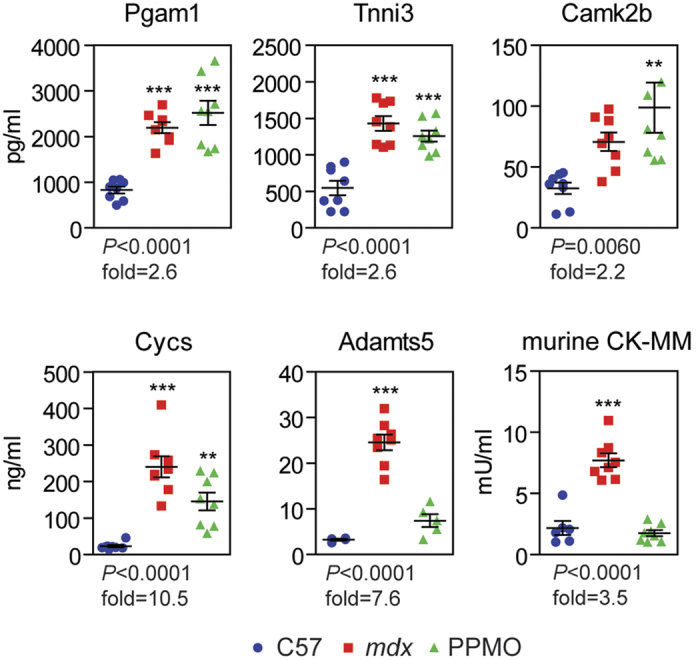
ELISA validation of candidate biomarkers. Five of the top candidate biomarker proteins were validated by ELISA using antibodies targeting the murine proteins. Murine CK-MM was included for comparison. Individual biological replicates are shown. Error bars indicate mean +/− SEM. One-way ANOVA *P* values and *mdx* vs C57 fold changes are indicated for each protein. Bonferroni *post hoc* test significance values are indicated as **P < 0.01, ***P < 0.001.

Several of the identified proteins have previously been reported as being elevated in dystrophic serum or as biomarkers of tissue damage in general (Table 1). We therefore selected a subset of proteins with no known previous association with DMD (Pgam1, Tnni3, Camk2b, Capn1 and Adamts5) and Cycs (which showed a strong response to therapy) for validation by ELISA (Fig. 3). Murine CK-MM was also included given its importance as an established DMD biomarker, although it ranked poorly relative to the top candidate proteins as determined by SOMAscan (Fig. 2, Table 1). Capn1 protein was not detectable by ELISA. Protein abundance was found to be significantly elevated in *mdx* serum (*P* < 0.0001) for all remaining candidates except for Camk2b (*P* < 0.06). Pgam1, Tnni3 and Camk2b were enriched in *mdx* serum by 2.2–2.6 fold (relative to wild-type controls) but showed little or no restoration towards wild-type levels after PPMO treatment. Clear shifts in protein abundance towards wild-type levels were observed for Adamts5, Cycs and CK-MM following PPMO treatment. The concentration of Cycs in *mdx* serum was increased by 11 fold in *mdx* serum and reduced to a 7 fold increase after exon-skipping treatment. Similarly, Adamts5 was enriched by 7.6 fold in *mdx* serum and restored to 2 fold after treatment. CK-MM levels were elevated by 4 fold in *mdx* serum and completely restored to wild-type mice levels following exon skipping therapy.

### ADAMTS5 is elevated in DMD, BMD and FSHD patient serum

Based on the SOMAscan data and ELISA validation we selected ADAMTS5 and PGAM1 for further investigation. ADAMTS5 exhibited the lowest *P* value in the SOMAscan screen (*P* = 0.000056) and profound restoration after therapy. Similarly, the ELISA data showed good normalisation after treatment with low inter-sample variation, thereby suggesting that ADAMTS5 may be useful as a biomarker for monitoring the response to therapy in DMD patients. The second lead candidate, PGAM1, showed only a mixed response to therapy but was the most elevated protein (136 fold) in *mdx* serum as determined by SOMAscan array.

**Figure 4:**
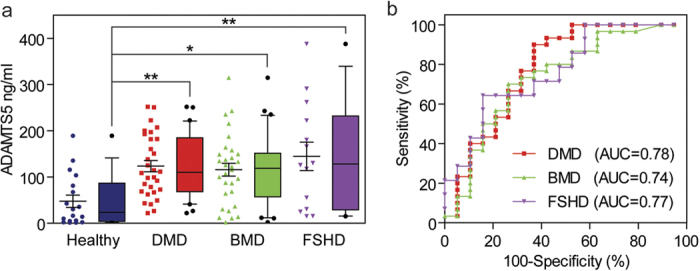
ADAMTS5 is elevated in DMD, BMD and FSHD patient serum. (**a**) ADAMTS5 abundance was measured by ELISA in serum samples from healthy control individuals (*n* = 18), DMD patients (*n* = 30), BMD patients (*n* = 30) and FSHD patients (*n* = 14). Data are shown as scatter plots showing individual replicates and box plots side-by-side. Error bars indicate mean +/− SEM. Box plots show median and interquartile range, whiskers represent the 10 to 90% range. Differences between groups were significant (*P* = 0.0023) as determined by one-way ANOVA. Bonferroni *post hoc* test significance values are indicated as **P < 0.01, *P < 0.05. (**b**) ROC curves for DMD, BMD and FSHD patients. AUC, Area Under the Curve.

Human-specific ELISAs for ADAMTS5 and PGAM1 were performed in DMD patient serum (*n* = 30) and compared with healthy controls (*n* = 18). Additionally, we also included serum from patients with Becker Muscular Dystrophy (BMD) (*n* = 30) and FacioScapuloHumeral muscular Dystrophy (FSHD) (*n* = 14). BMD is a dystrophinopathy with much milder symptoms than DMD^25^,^26^. In contrast, FSHD is an autosomal dominant muscular dystrophy caused by contraction of the D4Z4 macrosatellite repeat leading to toxic *DUX4* gain-of-function and therefore has a molecular pathogenesis that is distinct from DMD^27^. ADAMTS5 was significantly (*P* < 0.01) elevated in the serum of DMD patients by 3.4 fold relative to healthy controls (Fig. 4a). Similarly, ADAMTS5 was also elevated in BMD (*P* < 0.05) and FSHD (*P* < 0.01) patient serum (Fig. 4a). The mean of serum ADAMTS5 abundance was slightly lower in BMD patients relative to DMD although the median values were very similar. All experimental groups exhibited high variability, with the FSHD patient cohort being the most variable. Receiver operating characteristic (ROC) curve analysis showed that ADAMTS5 was similarly effective at discriminating between healthy and dystrophic individuals from each patient group (AUC ≥ 0.74, *P* < 0.01 in all cases) although the discrimination was marginally better for DMD (AUC = 0.78) (Fig. 4b). Consequently, ADAMTS5 may be useful for diagnosing muscle pathology or monitoring the response to therapy in patients with established diagnoses, but is not capable of distinguishing between DMD, BMD and FSHD pathology. No correlations were observed between ADAMTS5 levels and age in the case of the DMD or FSHD cohorts, whereas a negative correlation was observed for BMD (Supplementary Fig. S5). In contrast, PGAM1 was not detectable in any of the human serum samples.

## Discussion

Here we have utilised an aptamer-based proteomic screening approach to identify a plethora of novel candidate biomarkers for DMD. We successfully identified well-described biomarkers of generic muscle tissue damage (CK-MM, MB, LDHB, and FABP3)^28^,^29^,^30^ as being elevated in *mdx* sera (summarised in Table 1). Additionally, we also identified CYCS, TPI1 and THBS4 as being elevated in dystrophic serum, consistent with the findings of Hathout *et al.* utilising a mass spectrometry-based approach^22^ (Supplementary Table S1). All of these proteins were restored towards wild-type levels following dystrophin restoration, supporting their usefulness for monitoring the effectiveness of therapeutic interventions in DMD patients. Results for Pgam1, Tnni3, Camk2b, Cycs, Adamts5 and CK-MM were further assessed by ELISA. The proteins identified in the present study can be classified into a number of groups: (1) muscle function (MB, TNNI2, TNNI3), (2) metabolic dysregulation (PGAM1, LDHB, TPI1, FABP3), (3) calcium metabolism (CAMK2A, CAMK2B, CAMK2C, CAPN1), (4) extracellular matrix remodelling/fibrogenesis (ADAMTS5, THBS4), and (5) others. Interestingly, two proteins (TYMS and SFN) were less abundant in dystrophic serum, which cannot be easily explained as the result of passive leakage from damaged muscle.

Previous studies have investigated serum protein abundance in human DMD patients and dystrophic animal models using mass spectrometry^22^,^31^,^32^, bead-based antibody arrays^33^ or more focused approaches^34^,^35^,^36^. A limitation of the present study is that only the predetermined 1,129 proteins (for which there are SOMAmer reagents available) could be measured. However, this is still a greater number than has been detected by mass spectrometry (335 proteins detected)^22^ or by antibody-based bead arrays (315 target proteins)^33^. Notably, there were no SOMAmer probes targeted against many putative biomarker proteins identified in these studies (e.g. F13A1, FN1, TIMP1, MMP9, TNNT3, MYOM3), and so these were invisible to our study. Another potential limitation of our approach is that the SOMAmer target capture reagents were developed to bind to recombinant human proteins, and so interspecies differences in protein sequence may obscure important disease-associated changes. Similarly, cross-reactivity of SOMAmer reagents with proteins that are closely homologous with the target analyte may explain the discordant ELISA results for PGAM1 and CAPN1 (in these cases, it is possible that the SOMAmers cross-reacted with Pgam2^22^ and the muscle-specific calpain isoform Capn3 respectively). Additionally, some proteins may exist as processed fragments in the circulation^36^, or may form protein complexes, meaning that their target epitopes are absent or concealed. Together, these factors may explain some of the minor discrepancies between the SOMAscan data and ELISA validation. Notably, during the preparation of this manuscript Hathout *et al.* reported a SOMAscan study in DMD patient sera with many findings consistent with those presented here^37^ (Table 1, Supplementary Table S1). In this study, serum samples from 93 DMD patients and 45 control individuals was analysed using the SOMAscan platform and 44 significantly changed proteins were identified. Among these, TNNI2, TINN3, FABP3, MB, LDHB, AN32B and CAMK2A were consistent with the present study, thus confirming the robustness of the technique across human and mice. In contrast to our study, ADAMTS5 was not identified as being increased in DMD patient serum. This highlights a limitation of biomarker discovery studies in outbred patient populations where differences in genetic background may obscure important findings.

Consistent with our SOMAscan data, the concentration of ADAMTS5 was also found to be increased in DMD patient sera, supporting its use as a clinical biomarker. However, elevated ADAMTS5 was also observed in BMD and FSHD patients suggesting that it may be a non-specific marker of muscle pathology. Interestingly, expression of ADAMTS5 was previously shown to be elevated in the muscle of FSHD patients^38^. Notably, potential serum protein biomarkers for FSHD were recently identified in an antibody-based screen, although ADAMTS5 was not included in the panel of antibodies tested^39^.

In conclusion, we have identified multiple novel protein biomarkers in a murine model of DMD, one of which was validated in DMD patient serum. Many of these putative biomarkers were restored towards wild-type levels following dystrophin exon skipping, suggesting that these may be useful for monitoring the response to experimental therapies in clinical trials. In addition, protein-based serum biomarkers have the advantage that they can be easily measured with ELISA assays in an automated fashion and are therefore suitable for screening large patient cohorts. Several of the identified proteins are involved in DMD-associated pathophysiological processes such as metabolic dysfunction, loss of calcium homeostasis and fibrosis, suggesting that they may have utility as biomarkers for these specific features of DMD pathology. Future work will measure these biomarkers in larger patient cohorts, longitudinal studies, in patients treated with experimental therapies (e.g. exon skipping), and investigate the mechanisms which lead to secretion or leakage of these proteins from dystrophic muscle.

## Methods

### Animal Samples

All animal experimentation procedures were authorised and approved by the University of Oxford ethics committee and UK Home Office (project licence 30/2907) in accordance with the Animals (Scientific Procedures) Act 1986. 14 week old male C57/Bl10 and C57/Bl10ScSn-*Dmd*^*mdx*^/J (*mdx*) mice (*n* = 8) were sacrificed and blood was collected from the jugular vein using Microvette CB300 serum collection tubes (Sarstedt, Leicester, UK). Whole blood was allowed to clot on ice and then spun for 5 minutes at 10,000 *g*. 75 μl aliquots of serum were stored at −80 °C prior to analysis. 12 week old male *mdx* mice (*n* = 8) were administrated with a single dose of 12.5 mg/kg Pip6a-PMO conjugate prepared in a sterile saline solution via tail vein injection. Animals were sacrificed at 14 weeks of age and serum was harvested as described above. Pip6a-PMO consists of a PMO (5′-GGCCAAACCTCGGCTTACCTGAAAT) moiety chemically conjugated to an arginine-rich cell-penetrating peptide (Ac-RXRRBRRXR YQFLI RXRBRXRB-OH, where X is aminohexanoyl and B is β-alanine) and was synthesised and administered to the mice as described previously^1^,^12^.

### Human Samples

Serum samples from Duchenne Muscular Dystrophy (DMD), Becker Muscular Dystrophy (BMD) and FacioScapuloHumeral muscular Dystrophy (FSHD) patients were obtained from Newcastle University through the MRC Centre for Neuromuscular Diseases Biobank. Serum samples from healthy individuals were obtained from Newcastle University (as above, *n* = 5) or collected from volunteers at the University of Oxford (*n* = 13). All samples were collected according to standard operating procedures applied at both locations. Collection of samples from patients and their use in research have been ethically approved by the National Research Ethics Service (NRES) Committee North East – Newcastle and North Tyneside 1 in accordance with the Helsinki Declaration. Informed consent was obtained from all subjects.

### SOMAscan Serum Proteomics

Serum proteomics profiling was performed on the SOMAscan platform at SomaLogic, Inc. (Boulder, CO, USA). The SOMAscan platform measures the abundance of 1,129 target proteins, each of which is targeted by a unique SOMAmer reagent. Each SOMAmer consists of the protein binding aptamer component developed by SELEX (Systematic evolution of ligands by exponential enrichment), a photo-cleavable biotin moiety for the initial SOMAmer-protein complex capture step, and a Cyanine3 fluorophore for the purposes of detection and quantification. Three dilutions (0.5%, 2% and 5%) were prepared for each sample. Each sample dilution was separately mixed with a set of SOMAmer reagents that were immobilised on streptavidin-coated beads. Beads were washed to reduce non-specific protein binding. Proteins that remain captured on the SOMAmer-beads were biotinylated using NHS-PEO4-Biotin. After the labeling reaction, the beads are exposed to an anionic competitor solution that prevents non-specific interactions from reforming after they are disrupted^19^. Biotinylated-protein-SOMAmer complexes were released from the beads using ultraviolet light to cleave the photo-cleavable linker contained within the SOMAmer component. Eluates (containing SOMAmer-protein complexes and free SOMAmers) were incubated with streptavidin-coated beads for a second time to precipitate biotinylated-protein-SOMAmer complexes. Free SOMAmer reagents were removed by subsequent washing of the beads. Finally, protein-SOMAmer complexes were eluted using denaturing conditions and the nucleic acid (SOMAmer) component quantified by hybridization to custom DNA microarrays. Note: For convenience human Entrez gene identifiers are used to describe output from the SOMAscan analysis. The complete SOMAscan dataset is provided in Supplementary File S1.

### ELISA

Selected candidate proteins were validated using sandwich enzyme immunoassay. ELISA kits were purchased from antibodies-online (Aachen, Germany) and assay IDs are listed in Supplementary Table S2. Whenever possible, sera of the animals used for the SOMAscan screen was used. Due to limited amount of sample, additional age- and sex-matched animals were included in the study so that each group consisted of eight samples. ELISAs were performed according to manufacturer’s instructions and the serum was diluted to fall within the linear range of each respective assay. Sample concentrations were extrapolated with GraphPad Prism 5 (GraphPad Software Inc, La Jolla, CA) using fourth-order polynomial data fit of the standard curves.

### Statistical Analysis

SOMAscan data were analysed using the SOMAsuite analysis software to perform Mann-Whitney U test (two-sided) and Kruskal-Wallis one-way ANOVA. Non-parametric analyses were used as SOMAscan data were determined to not be normally distributed as determined by Shapiro-Wilk test (GraphPad Prism 5). Hierarchical clustering and heatmap visualization was performed using MeV (Multiple Experiment Viewer (The Institute for Genomic Research, Rockville, MD, USA)^40^. Principal component analysis was performed in R version 3.2. Additional statistical functions were performed in GraphPad Prism 5: one-way ANOVA, Bonferroni *post hoc* test, Pearson/Spearman correlation, linear regression and ROC curve analysis.

## Additional Information

**How to cite this article**: Coenen-Stass, A. M. L. *et al.* Identification of novel, therapy-responsive protein biomarkers in a mouse model of Duchenne muscular dystrophy by aptamer-based serum proteomics. *Sci. Rep.* **5**, 17014; doi: 10.1038/srep17014 (2015).

## Supplementary Material

Supplementary Information

## Figures and Tables

**Table 1:** Top candidate serum protein biomarkers for DMD identified by SOMAscan.

Entrez ID Name	UniProt ID	Fold Change (*mdx* vs C57)	Dilution (%)	Restoration by PPMO	Reference
**PGAM1** Phosphoglycerate mutase 1	P18669	136	5	++	
**TNNI3** Troponin I, cardiac muscle	P19429	53	5	+++	37
**MB** Myoglobin	P02144	15	2	++	21,22,37
**TNNI2** Troponin I, fast skeletal muscle	P48788	14	5	+	37
**LDHB** L-lactate dehydrogenase B chain	P07195	8.4	0.5	++	23,37
**FABP3** Fatty acid-binding protein, heart	P05413	7.8	5	++	22,37
**TPI1** Triosephosphate isomerase	P60174	5.7	2	-	22
**CAMK2B** Calcium/calmodulin-dependent protein kinase type II subunit beta	Q13554	5.3	5	++	
**CAMK2A** Calcium/calmodulin-dependent protein kinase type II subunit alpha	Q9UQM7	4.1	5	++	37
**ANP32B** Acidic leucine-rich nuclear phosphoprotein 32 family member B	Q92688	3.7	2	++	37
**CYCS** Cytochrome c	P99999	3.6	2	+++	22
**CAMK2D** Calcium/calmodulin-dependent protein kinase type II subunit delta	Q13557	3.6	5	++	
**PCNA** Proliferating cell nuclear antigen	P12004	3.3	5	++	
**HTRA2** Serine protease HTRA2, mitochondrial	O43464	3.1	5	+++	
**ADAMTS5** A disintegrin and metalloproteinase with thrombospondin motifs 5	Q9UNA0	2.8	5	+++	
**PTPN11** Tyrosine-protein phosphatase non-receptor type 11	Q06124	2.8	5	++	
**LYN** Tyrosine-protein kinase Lyn	P07948	2.7	5	++	
**DUSP3** Dual specificity protein phosphatase 3	P51452	2.5	5	—	
**CAPN1** Calpain I	P07384 P04632	2.4	2	+++	
**THBS4** Thrombospondin-4	P35443	2.2	2	++	22
**EDA2R** Tumor necrosis factor receptor superfamily member 27	P35443	2.1	5	++	
**TYMS** Thymidylate synthase	P04818	−2.7	5	++	
**SFN** 14-3-3 protein sigma	P31947	−3.2	2	++	

+++restored to wild-type levels, ++ restored towards wild-type levels, + inconsistent restoration towards wild-type levels between replicates, —not restored towards wild-type levels.
