# Corrigendum: Identification of novel, therapy-responsive protein biomarkers in a mouse model of Duchenne muscular dystrophy by aptamer-based serum proteomics

**DOI:** 10.1038/srep37047

**Published:** 2016-12-19

**Authors:** Anna M. L. Coenen-Stass, Graham McClorey, Raquel Manzano, Corinne A. Betts, Alison Blain, Amer F. Saleh, Michael J. Gait, Hanns Lochmüller, Matthew J. A. Wood, Thomas C. Roberts

Scientific Reports 5: Article number: 1701410.1038/srep17014; published online: 11 23 2015; updated: 12 19 2016

Supplementary Dataset S1 was omitted from the original version of this Article. This error has now been corrected.

