# Supplementary material for: Identification of novel, therapy-responsive protein biomarkers in a mouse model of Duchenne muscular dystrophy by aptamer-based serum proteomics
Source: Sci Rep. 2015 Nov 23;5:17014. doi: 10.1038/srep17014 (PMC4655324; doi:10.1038/srep17014)
Supplement: Supplementary Information [file srep17014-s1.pdf]

Supplementary Information

Title

Identification of novel, therapy-responsive protein biomarkers in a mouse model of Duchenne muscular dystrophy by aptamer-based serum proteomics

Authors

Anna M. L. Coenen-Stass[1], Graham McClorey[1], Raquel Manzano[1], Corinne A. Betts[1], Alison Blain[2], Amer F. Saleh[3,4], Michael J. Gait[3], Hanns Lochmüller[2], Matthew J. A. Wood[1], Thomas C. Roberts[1,5]

Author Affiliations

[1]Department of Physiology, Anatomy and Genetics, University of Oxford, South Parks Road, Oxford, OX1 3QX, UK

[2]The John Walton Muscular Dystrophy Research Centre, MRC Centre for Neuromuscular Diseases, Institute of Genetic Medicine, Newcastle University, Central Parkway, Newcastle upon Tyne, NE1 3BZ, UK

[3]Medical Research Council, Laboratory of Molecular Biology, Francis Crick Avenue, Cambridge, CB2 0QH, UK

[4]Current address: AstraZeneca R&D, Discovery Safety, Drug Safety and Metabolism, Alderley Park, Macclesfield, SK10 4TG, UK

[5]Sanford Burnham Prebys Medical Discovery Institute, Development, Aging and Regeneration Program, La Jolla, CA, 92037, USA

**Rouillon *et al.*** [1]	**Hathout *et al.*** [2]	**Ayoglu *et al.*** [3]	**Hathout *et al.*** [4]	**Coenen-Stass *et al.* (present study)**	**Alagaratnam *et al.*** [5]
**MS**	**MS**	**Ab array**	**SOMAscan**	**SOMAscan**	**MS**
GOT1	ACTA1	CA3	ACY1	ADAMTS5	F13A1
GPT	ADIPOQ	CKM	ADAM9	ANP32B	
ALDOA	ALDOA	COL6A1	**ANP32B**	CAMK2A	**Nadarajah *et al.*** [6]
IGFALS	CKM	ENO3	CA3	CAMK2B	**ELISA**
C4BPB	**CYCS**	ETFA	**CAMK2A**	CAMK2D	MMP9
CADH5	ENO3	ETFB	CD55	CAPN1	
CA3	**FABP3**	LCP1	CDH5	CYCS	
CD109	FIBG	MDH2	CHL1	DUSP3	
CETP	FLNC	MYL3	CKM	EDA2R	
CHL1	GAPDH	PPM1F	CNTN5	FABP3	
CNDP1	PKM	TNNT3	CXCL10	HTRA2	
COLEC11	LDHA		**FABP3**	LDHB	
CRAC1	LIFR		FAP	LYN	
DPP4	LUM		FGA/FGB/FGG	MB	
ENO3	MDH1		GDF11	PCNA	
FLNC	**MB**		GPI	PGAM1	
GPI	MYH4		GPT	PTPN11	
HBA1/2	MYL1		GSN	SFN	
HBB	MYOM3		HDGFRP2	THBS4	
HBD	PGAM2		HSPA1A	TNNI2	
HP	PLG		IBSP	TNNI3	
CKM	PVALB		IL34	TPI1	
PKM	PYGM		JAG1	TYMS	
LBP	SAA2		**LDHB**		
LDHA	TTN		MAPK12		
**LDHB**	TPI1		**MB**		
**MB**	**THBS4**		MDH1		
MYH7			NOTCH3		
MYOM2			OMD		
MYOM3			PLA2G2A		
GPLD1			PRKACA		
PYGM			PSMA2		
TTN			PSPN		
**TPI1**			RELT		
TPM2			RET		
VASN			RPS7		
VCL			SERPIND1		
			SET		
			SPINT1		
			**TNNI2**		
			**TNNI3**		
			UNC5C		
			WFIKKN1		
			WIF1		

Supplementary Table S1

Comparison of key DMD serum biomarkers findings across seven studies.

Methodologies used in each study are indicated. Proteins for which there was no SOMAmer detection reagent (and were therefore invisible to the SOMAscan methodology) are highlighted in red. Protein biomarker candidates that were commonly detected with the present study are highlighted in bold.

**Mouse**	**Product code**	**Sensitivity**	**Comments**
CALPN1	ABIN424286	1.56-100ng/ml	Sample below detection limit
CYCS	ABIN1569298	1.56-100ng/ml	
PGAM1	ABIN820296	0.025-1.6 ng/ml	
Troponin I	ABIN853281	0.0156-1.0 ng/ml	
ADAMTS-5	ABIN827175	0.625-40 ng/ml	
CAMK2B	ABIN811938	0.03125-2.0 ng/ml	
CK-MM	ABIN1000121	0.156-10 mU/mL	
**Human**			
ADAMTS-5	ABIN649163	0.313-20 ng/mL	
PGAM1	ABIN820295	28.12-1800 pg/mL	Samples below detection limit

Supplementary Table S2

Details of ELISA assays used in this study.

All ELISA assays were purchased from Antibodies Online.

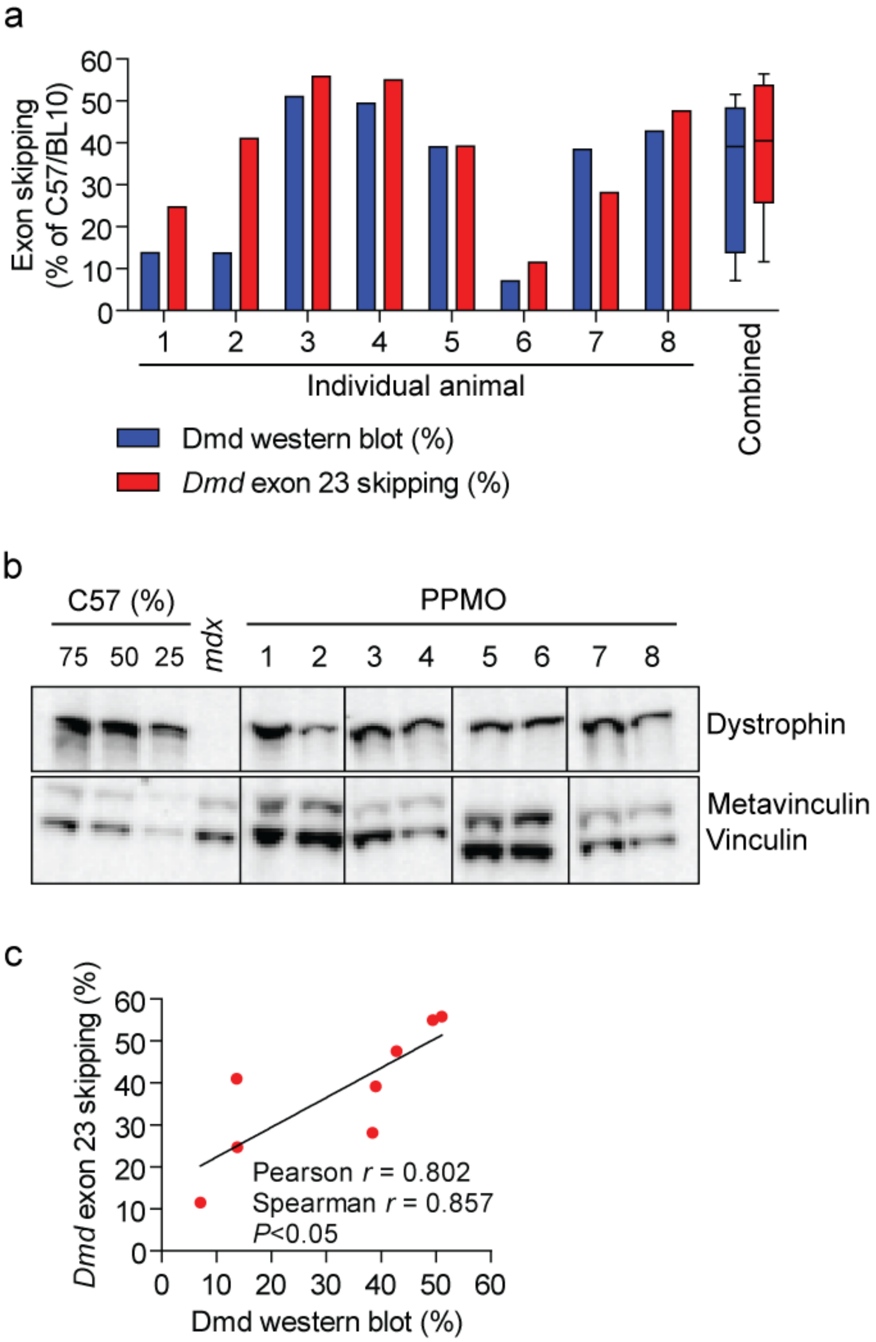
a
Exon skipping (% of C57/BL10)
0
10
20
30
40
50
60
1
2
3
4
5
6
7
8
Individual animal
Combined
Dmd western blot (%)
Dmd exon 23 skipping (%)
b
C57 (%)
75
50
25
mdx
PPMO
1
2
3
4
5
6
7
8
Dystrophin
Metavinculin
Vinculin
c
Dmd exon 23 skipping (%)
Dmd western blot (%)
Pearson *r* = 0.802
Spearman *r* = 0.857
P<0.05

Supplementary Figure S1

Quantification of Pip6a-PMO therapeutic efficacy.

(a) Efficiency of Pip6a-PMO therapy was assessed in quadriceps femoris muscles by western blot to measure dystrophin protein and RT-qPCR to detect the degree of *Dmd* exon 23 skipping. Values for each individual animal are shown along with Tukey box plots of the combined data. (b) Images of western blots. (c) *Dmd* exon 23 skipping and dystrophin protein expression were positively correlated.

a

Group	Sample	Hybridization Normalization Scale Factor	Median Normalization Scale Factor		
			**5%**	**2%**	**0.5%**
**C57**	C1	0.938	0.938	0.913	0.953
	C2	0.913	1.081	1.092	1.1
	C3	0.962	1.002	0.997	0.878
	C4	0.94	1.024	1.01	1.081
	C5	0.94	0.927	0.935	0.956
	C6	0.929	1.09	1.093	1.099
	C7	0.917	1.076	1.101	1.004
	C8	0.966	1.041	1.077	1.022
mdx	M1	0.908	1.007	0.993	0.995
	M2	0.886	0.813	0.837	0.92
	M3	0.948	0.981	0.946	0.943
	M4	0.924	0.852	0.857	0.818
	M5	0.932	0.884	0.869	0.937
	M6	0.969	0.955	0.95	0.964
	M7	0.956	0.842	0.831	0.827
	M8	0.93	0.938	0.925	0.852
**PPMO**	T1	0.91	1.146	1.153	1.138
	T2	0.966	1.008	1.026	1.063
	T3	0.924	1.063	1.09	1.023
	T4	0.96	0.932	0.995	1.003
	T5	0.944	1.08	1.1	1.165
	T6	0.952	1.023	1.01	1.026
	T7	0.979	**6.105**	**7.026**	**3.869**
	T8	0.931	1.124	1.108	1.091

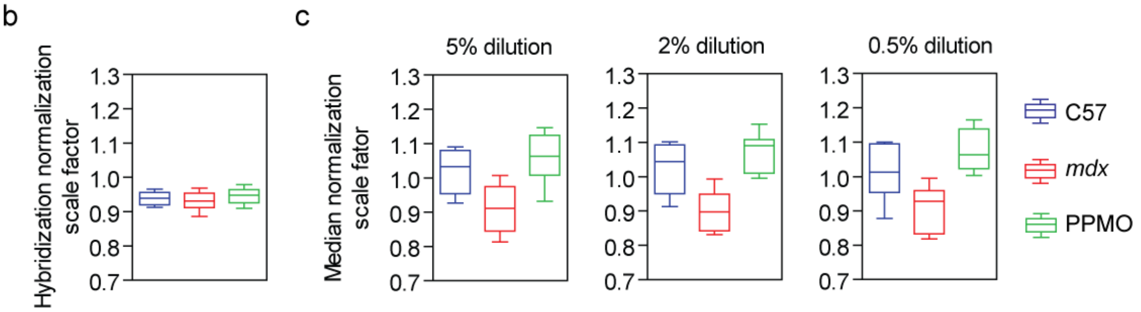

Supplementary Figure S2

Quality control of SOMAscan data.

(a) Table of all hybridization normalization and median normalization (at all three dilutions) scale factors. The acceptable range is 0.4-2.5. All scale factors were within the range and generally close to 1 (i.e. no scaling) with the exception of sample T7 (labelled in red) – which was excluded from subsequent analyses. (b) Box plots of hybridization normalization scale factors separated by experimental group. (c) Box plots of median normalization scale factors at all three dilution separated by experimental group. Scale factors were generally lower in *mdx* samples indicating a gross increase in protein content.

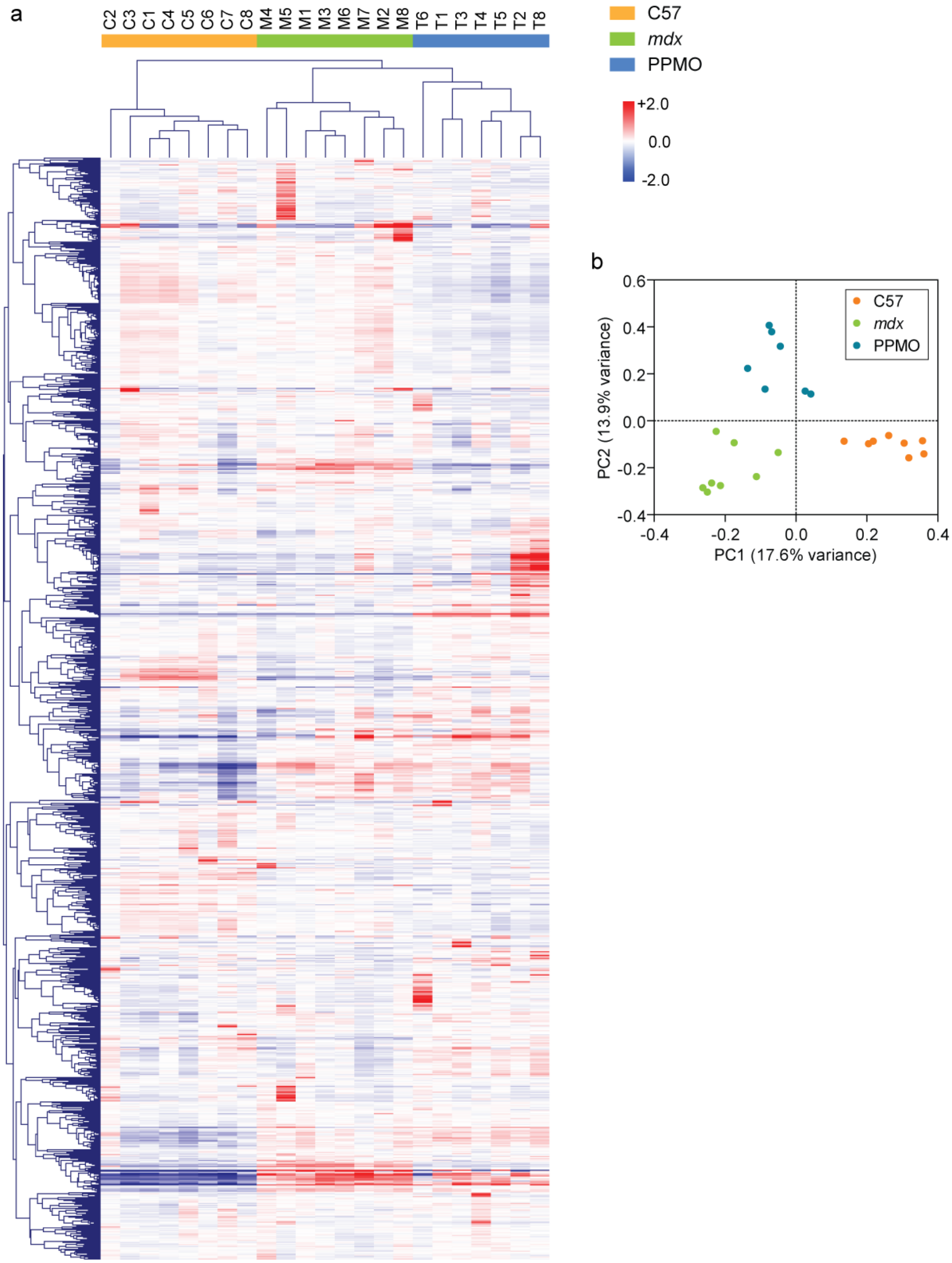
a
C2 C3 C1 C4 C5 C6 C7 C8 M4 M5 M1 M3 M6 M7 M2 M8 T6 T1 T3 T4 T5 T2 T8
C57
mdx
PPMO
+2.0
0.0
-2.0
b
C57
mdx
PPMO
PC1 (17.6% variance)
PC2 (13.9% variance)
0.6
0.4
0.2
0.0
-0.2
-0.4
-0.4
-0.2
0.0
0.2
0.4

Supplementary Figure S3

Unsupervised analyses of serum protein expression.

(a) All protein detection signals were analysed by unsupervised hierarchical clustering and (b) principal components analysis (the first two components representing 31.4% of the data are shown). Red indicates up-regulated proteins and blue indicates down-regulated proteins. The scale bar represents the row Z-score.

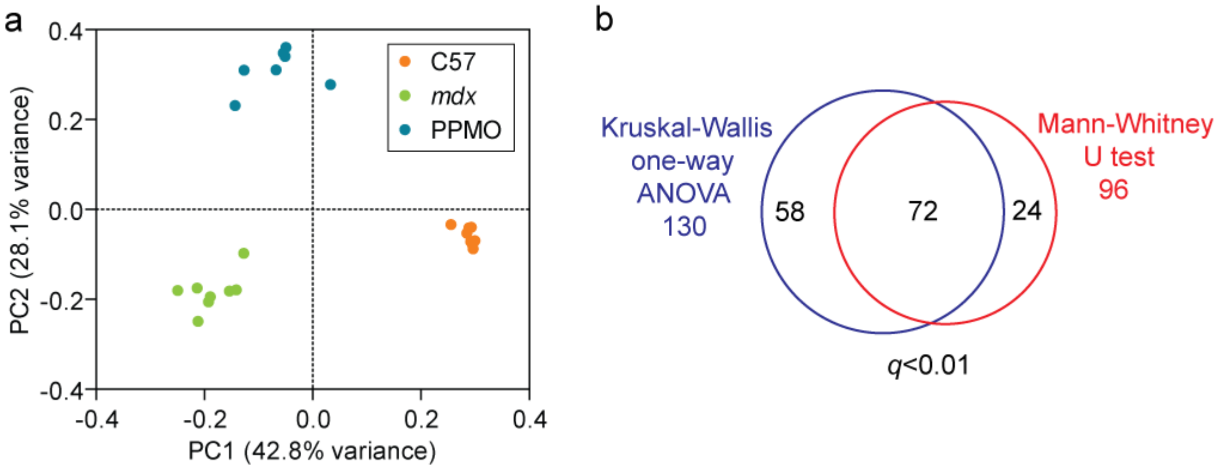
a
b
0.4
0.2
0.0
-0.2
-0.4
-0.4
-0.2
0.0
0.2
0.4
PC2 (28.1% variance)
PC1 (42.8% variance)
C57
mdx
PPMO
Kruskal-Wallis one-way ANOVA 130
Mann-Whitney U test 96
58
72
24
q<0.01

Supplementary Figure S4

Statistical analysis of SOMAscan data.

(a) Statistically significant (P<0.01) protein changes were visualized by principal component analysis (the first two components representing 71% of the data are shown). (b) A high degree of overlap was observed between statistically significant protein calls from both Mann-Whitney U test and Kruskal-Wallis one-way ANOVA analyses.

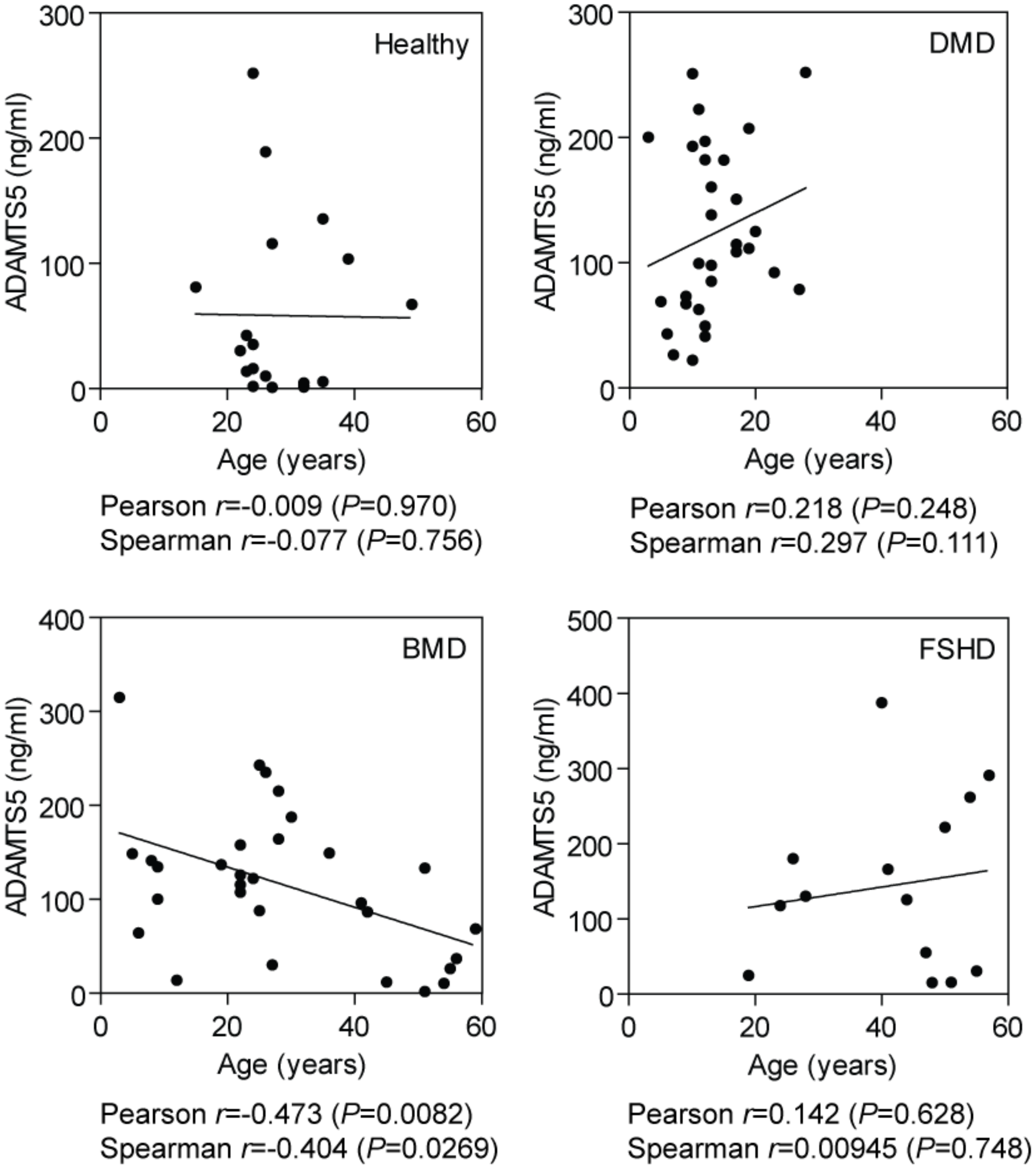

Healthy
ADAMTS5 (ng/ml)
Age (years)
Pearson r=-0.009 (P=0.970)
Spearman r=-0.077 (P=0.756)
DMD
ADAMTS5 (ng/ml)
Age (years)
Pearson r=0.218 (P=0.248)
Spearman r=0.297 (P=0.111)
BMD
ADAMTS5 (ng/ml)
Age (years)
Pearson r=-0.473 (P=0.0082)
Spearman r=-0.404 (P=0.0269)
FSHD
ADAMTS5 (ng/ml)
Age (years)
Pearson r=0.142 (P=0.628)
Spearman r=0.00945 (P=0.748)

Supplementary Figure S5

Correlation plots of serum ADAMTS5 and age.

For each patient/healthy group, the ADAMTS5 ELISA results were plotted against age. A statistically significant negative correlation was observed for the BMD patient group only.

Supplementary Methods

RT-qPCR

RNA was extracted from quadriceps cryo-sections using TRIzol Reagent (Life Technologies). 1 µg of RNA was reverse transcribed using the High Capacity cDNA Kit (Life Technologies) according to manufacturer's instructions. 25 ng of cDNA template was amplified for each sample on a StepOne Plus real-time PCR Thermocycler (Life Technologies) using universal cycling conditions. PCR reactions consisted of TaqMan Gene Expression Master Mix (Life Technologies) and primer/probe assays in multiplex format. Levels of *Dmd* exon 23 skipping was determined using FAM-assay spanning the exon 20-21 boundary and a HEX-assay spanning the exon 23-24 boundary (Assay IDs: Mm.PT.47.9564450 and Mm.PT.47.7668824 respectively, Integrated DNA Technologies, Leuven, Belgium). The percentage of *Dmd* transcripts lacking exon 23 was determined by normalising exon 23-24 amplification levels to exon 20-21 levels.

Western Blot

Quadriceps femoris muscles were macrodissected and snap frozen in liquid nitrogen-cooled isopentane. 8 µm cryosections were prepared from the mid-belly of the muscle and samples lysed in buffer (75 mM Tris–HCl pH 6.5, 10% sodium dodecyl sulphate, 5% 2-mercaptoethanol and protease inhibitors) prior to centrifuging at 13,000 rpm for 10 minutes to pellet debris. Supernatants were collected and incubated at 100 ^{o}C for 3 minutes. Protein lysates were separated on a 3-8% Tris-Acetate gel (Life Technologies), electrotransferred to PVDF membrane and probed with monoclonal anti-dystrophin (1:200, NCL-DYS1, Novocastra) and anti-vinculin (loading control, 1:100,000, hVIN-1, Sigma) primary antibodies as previously described [7]. Secondary antibody IRDye 800CW goat anti-mouse was used at a dilution of 1:20,000 (LiCOR).

Fluorescence was detected and quantified using the Odyssey imaging system. Dystrophin expression was quantified using the dystrophin to vinculin ratio compared with C57/BL10 wild-type dystrophin expression standards on each gel.

Supplementary References

1. Rouillon, J. *et al.* Serum proteomic profiling reveals fragments of MYOM3 as potential biomarkers for monitoring the outcome of therapeutic interventions in muscular dystrophies. *Hum. Mol. Genet.* ddv214 (2015). doi:10.1093/hmg/ddv214
2. Hathout, Y. *et al.* Discovery of serum protein biomarkers in the mdx mouse model and cross-species comparison to Duchenne muscular dystrophy patients. *Hum. Mol. Genet.* **23,** 6458–6469 (2014).
3. Ayoglu, B. *et al.* Affinity proteomics within rare diseases: a BIO-NMD study for blood biomarkers of muscular dystrophies. *EMBO Mol Med* (2014). doi:10.15252/emmm.201303724
4. Hathout, Y. *et al.* Large-scale serum protein biomarker discovery in Duchenne muscular dystrophy. *Proc. Natl. Acad. Sci. U.S.A.* (2015). doi:10.1073/pnas.1507719112
5. Alagaratnam, S. *et al.* Serum protein profiling in mice: identification of Factor XIIIa as a potential biomarker for muscular dystrophy. *Proteomics* **8,** 1552–1563 (2008).
6. Nadarajah, V. D. *et al.* Serum matrix metalloproteinase-9 (MMP-9) as a biomarker for monitoring disease progression in Duchenne muscular dystrophy (DMD). *Neuromuscul. Disord.* **21,** 569–578 (2011).
7. Roberts, T. C. *et al.* Extracellular microRNAs are dynamic non-vesicular biomarkers of muscle turnover. *Nucl. Acids Res.* **41,** 9500–9513 (2013).
